# Lipid remodeling of contrasting maize (*Zea mays* L.) hybrids under repeated drought

**DOI:** 10.3389/fpls.2023.1050079

**Published:** 2023-05-10

**Authors:** Markus Kränzlein, Sandra M. Schmöckel, Christoph-Martin Geilfus, Waltraud X. Schulze, Michael Altenbuchinger, Holger Hrenn, Ute Roessner, Christian Zörb

**Affiliations:** ^1^ Institute of Crop Science, University of Hohenheim, Stuttgart, Germany; ^2^ Department of Soil Science and Plant Nutrition, Geisenheim University, Geisenheim, Germany; ^3^ Department of Plant Systems Biology, University of Hohenheim, Stuttgart, Germany; ^4^ Department of Medical Bioinformatics, University Medical Center Göttingen, Göttingen, Germany; ^5^ Core Facility Hohenheim, University of Hohenheim, Stuttgart, Germany; ^6^ Research School of Biology, The Australian National University, Acton, ACT, Australia

**Keywords:** maize, repeated drought stress, recovery, drought resistance, metabolomics

## Abstract

The role of recovery after drought has been proposed to play a more prominent role during the whole drought-adaption process than previously thought. Two maize hybrids with comparable growth but contrasting physiological responses were investigated using physiological, metabolic, and lipidomic tools to understand the plants’ strategies of lipid remodeling in response to repeated drought stimuli. Profound differences in adaptation between hybrids were discovered during the recovery phase, which likely gave rise to different degrees of lipid adaptability to the subsequent drought event. These differences in adaptability are visible in galactolipid metabolism and fatty acid saturation patterns during recovery and may lead to a membrane dysregulation in the sensitive maize hybrid. Moreover, the more drought-tolerant hybrid displays more changes of metabolite and lipid abundance with a higher number of differences within individual lipids, despite a lower physiological response, while the responses in the sensitive hybrid are higher in magnitude but lower in significance on the level of individual lipids and metabolites. This study suggests that lipid remodeling during recovery plays a key role in the drought response of plants.

## 1 Introduction

With drought being a major challenge in modern agriculture, a better understanding of drought adaptation and tolerance mechanisms is crucial for the breeding of more tolerant maize lines. While plant responses to singular stress events are well understood, responses toward repeated stress events have recently received increased attention, as the ability to recover from stress might have a bigger impact on total adaptability than previously thought (Rekowski et al., 2021; Schulze et al., 2021). The time after the first stimulus is thought to be a crucial phase in which plants may retain metabolic signals of the stress event (retaining imprints), or they return to a phenological prestressed state to restart and maximize growth (recovery). The phase in which signals are retained is referred to as stress memory or stress imprint (Walter et al., 2011; Crisp et al., 2016; Wedeking et al., 2018; Hilker & Schmülling, 2019). These different strategies (retaining a memory vs. recovery) are viable under different environmental conditions; if the poststress environment is characterized by shorter, less severe stress events, the recovery-oriented strategy might be advantageous, while the memory strategy might be better at dealing with longer, more severe upcoming stress periods that are intermitted by longer periods of ambient conditions (Skirycz & Inzé, 2010; Crisp et al., 2016). It has been observed that plants that had experienced a non-lethal initial drought stress are able to survive a subsequent severe drought stress (Crisp et al., 2016). This has been attributed to the formation of a stress memory after the initial drought treatment. The mechanisms associated with maintaining stress memory require careful regulation as they are likely to be energy intensive, thus resulting in reduced growth and yield (Huot et al., 2014). Crop plants that have been bred for higher yield formation may have lost some of the genetic variation related to stress memory formation (Tanksley & McCouch, 1997). Therefore, in addition to yield formation, plant breeding targets now also include traits for resilience to drought stress (Reynolds et al., 2021).

In the context of drought adaptation, lipids have received much more attention in the recent decade, showing that plant lipids are crucial for energy metabolism, stress signaling (Hou et al., 2016), growth, and development (Fujii et al., 2014; Kobayashi, 2016). The cell membrane is comprised of lipids, which are prone to oxidative processes, that are enhanced under drought stress by the production of reactive oxygen species (ROS) in cellular organs like mitochondria, peroxisomes, and chloroplasts. However, beneficial signaling characteristics of ROS in response to stresses, like the activation of defense- and recovery-related genes, are also known (Triantaphylidès and Havaux, 2009; Farmer and Mueller, 2013; Huang et al., 2019). Membranes and cellular structures can be protected from the deleterious effects of ROS by an increased antioxidant capacity, or by counteracting through lipid remodeling, such as the modulation of membrane fluidity, the accumulation of triacylglycerol (TG) for sequestering released cytotoxic free fatty acids and diacylglycerol (DG), leading to the formation of lipid droplets (Liu et al., 2019; Yu et al., 2021). The major phospholipids in the plasma membrane (PM) are the bilayer lipid phosphatidylcholine (PC) and the non-bilayer lipid phosphatidylethanolamine (PE), as well as phosphatidylinositol (PI) and phosphatidylserine (PS) (Yu et al., 2021). The main lipids of the thylakoid membrane are the galactolipids monogalactosyl-diacylglycerol (MGDG), digalactosyl-diacylglycerol (DGDG), sulfoquinovosyldiacylglycerol (SQDG), and the phospholipid phosphatidylglycerol (PG) (Joyard et al., 1998; Dörmann and Benning, 2002; Kobayashi, 2016). The different shapes of the headgroups (the conic shape of MGDG and the cylindric shape of DGDG and SQDG) (Shipley et al., 1973) are responsible for the grana stacking (Demé et al., 2014) and for the functioning of photosynthesis (Williams, 1998; Wang and Benning, 2011). Under oxidative stress, the highly unsaturated chains of the galactolipids are oxidized (Ferrari-Iliou et al., 1994), causing a disruption of membrane fluidity and photosynthesis. The plant may adapt by increasing the ratio of DGDG/MGDG (Gigon et al., 2004; Shimojima and Ohta, 2011), such that MGDG is downregulated and mainly converted into DGDG and thus stabilize grana stacking (Gasulla et al., 2013). It has also been discussed that the unsaturated chains of the galactolipids serve as scavengers of singlet oxygen, the primary source of ROS in the plastid (Farmer and Mueller, 2013). The increased saturation of galactolipids under abiotic stress is part of membrane lipid remodeling and has been shown to stabilize membrane fluidity and photosynthesis (Sui et al., 2010). It is known that lipid degradation is one of the first responses to water deficit, as the activity of phospholipases and other lipolytic enzymes increases (Sahsah et al., 1998). Furthermore, the reduced cellular water content contributes to a disruption of membrane fluidity and protein interactions, with the chloroplast membrane being the first target for degradation, leading to premature leaf senescence (Quirino et al., 2000; Guo and Gan, 2005). This premature leaf senescence has been shown to be delayed in genotypes showing an increased DGDG/MGDG ratio and higher lipid unsaturation under drought stress in maize (Chen et al., 2018). Even though lipids clearly occupy a central role in stress adaptation, only few studies investigate changes of the lipid profile under repeated stress or dynamic environments.

In this study, we compared two maize hybrids with contrasting physiological and lipid responses to repeated drought. We conclude that contrasting lipid remodeling patterns may account for a large portion of the different sensitivities of the maize hybrids to drought stress, which is consistent with differences in ion leakage in response to drought. Moreover, the recovery phase turned out to be the most crucial phase that decides overall drought tolerance in the upcoming second drought stress in these contrasting hybrids.

## 2 Materials and methods

### 2.1 Plant material and cultivation

Two maize hybrids with contrasting resilience to repeated drought were used based on a previous experiment (Kränzlein et al, 2022). Hybrid L (‘LG30222’) is considered tolerant to drought, and hybrid K (‘KWS stabil’) is considered more sensitive to drought. A total of 35 seeds per hybrid were soaked for 24 h with aerated 1 mM CaSO_4_. The day after, on 26 June 2019, the pretreated seeds were planted in 7 L pots, each containing 4.3 kg of a 1:1 subsoil/sand-substrate (v/v) mixture. The experiment was conducted in a greenhouse at the University of Hohenheim for a period of 41 d. After each phase of the experiment (see Section 2.2) on d 31, 35, and 41, five plants per treatment and hybrid were harvested (n = 5). During the experiment, the pots were randomly rearranged once per week to reduce positional effects. Liquid fertilizer (KH_2_PO_4_, NH_4_NO_3_, MgSO_4_*7 H_2_O) was applied once per week; the total amount of nutrients applied was 1.25 g N, 0.5 g K, 0.4 g P, 0.68 g Mg, 0.9 g S.

### 2.2 Stress treatment

**Figure 1:**
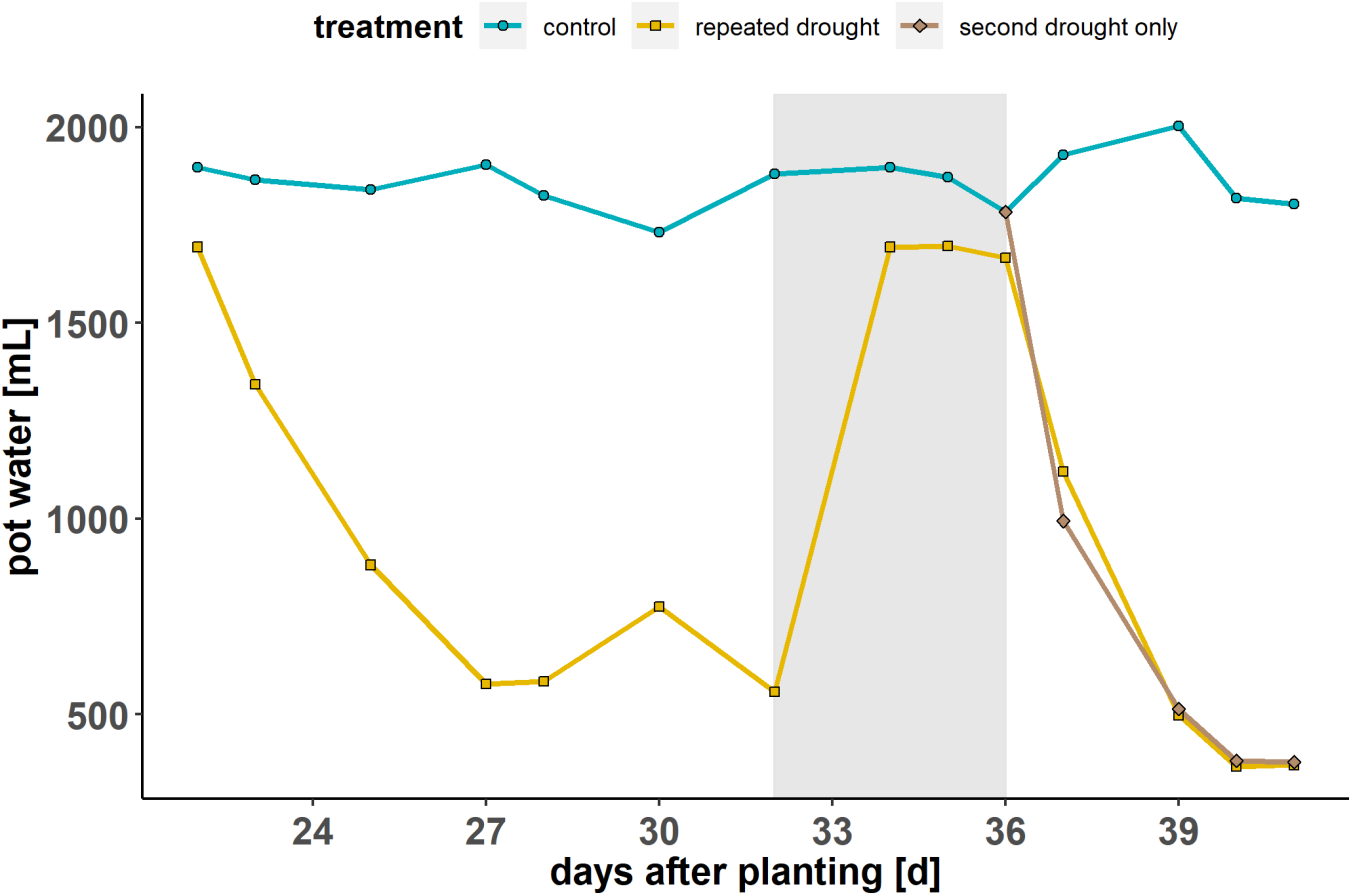
Experimental overview: soil-substrate water content during the phases of the experiment (first drought—recovery—second drought only) for the three treatment groups (control—repeated drought—second drought only). The gray background indicates the recovery phase during rewatering. Modified from Schulze et al. (2021).

The maximum water-holding capacity (WHC) of the substrate-sand mixture was determined at the beginning of the experiment. The WHC of the control conditions was set to 60 %, which corresponds to well-watered conditions based on a pre-experiment using the same hybrids and a similar substrate-sand mixture (Kränzlein et al, 2022). The WHC in the experiment was controlled using weight measurements of the pots at least once per day (**Figure 1**). The plants were exposed to three treatments: control condition (continuously 60 % WHC), repeated drought treatment (first drought 17 % WHC, second drought ~11 % WHC) and a second drought–only treatment (control conditions until the second drought, then ~12 % WHC). The repeated drought treatment was ~17 % WHC (mild drought) for 1 week, followed by a 4 d recovery period and a second, terminal drought treatment for 4 d, reaching ~11 % WHC (severe drought).

### 2.3 Leaf elongation and gas exchange measurements

During the greenhouse experiment, non-destructive measurements including the leaf elongation rate (LER) and gas exchange were measured every 1–2 days (see the timeline in **Figure 1**). For LER measurements, the length of the leaf from the surface of the pot to the tip was recorded for all growing leaves. The LER was then expressed in cm h^-1^ as a mean of the LER of all the elongating leaves per time point. Gas exchange measurements of the assimilation rate (A) and stomatal conductance (*gs*) were conducted on leaves 6 and 7 (counted from the plant base) throughout the experiment using a LiCl device (ADC BioScientific Ltd., Hoddesdon, England). The same area of leaf 6 and 7 was used for the gas exchange measurements.

### 2.4 Sampling of plant material for ion leakage, osmotic pressure measurements, metabolomics, and lipidomics

For ion leakage measurements, osmolality, and metabolomics analyses, fully elongated leaves from leaf 6 and 7 were sampled: leaf disks for ion leakage measurements were taken using a cork borer (0.75 cm diameter); then, the middle vein of the remaining leaf was removed, and a subset of the leaf was prepared for osmolality measurements by squeezing the leaf material using a handheld press and freezing the sap immediately at -20 °C until the osmolality measurements were taken. The remaining leaf material was frozen in liquid nitrogen for metabolomics analyses. The rest of the plant material that was not used for ion leakage; osmolality and metabolomics analyses were weighted immediately at harvest to record the fresh weight (FW) of the shoots. The root material was washed with deionized water and dried in an oven at 50 °C for 6 h, and the root dry weight (DW) was recorded.

#### 2.4.1 Measuring ion leakage and osmolality

For ion leakage measurements, 10 leaf disks per plant were excised, rinsed for 3 s with 18 MΩ water, and then transferred to a 50 ml centrifugation tube filled with 15 ml of 18 MΩ water. The tubes were gently shaken for 5 h. The conductivity was measured using a conductometer (WTW LF90; WTW KLE1 cell, Weilheim, Germany). The tubes were frozen overnight and then thawed at room temperature until the solution had equilibrated to room temperature. The final conductivity was recorded afterward. Total conductivity was then expressed as the ratio of conductivity after 5 h and the total conductivity after thawing. The frozen samples for osmolality measurements were thawed and centrifuged at 11,000 g for 10 min; then, the osmolality of the supernatant was measured with a vapor-pressure osmometer (VAPRO^®^ Vapor Pressure Osmometer, ELITechGroup, Paris, France) three times per sample.

### 2.5 Primary metabolite profiling

The frozen leaf material was freeze-dried and ground using a mixer mill (Retsch, Germany). Pulverized samples were used for lipidomics and metabolomics. 25 mg of the freeze-dried sample was weighted into a 1.5 ml reaction tube; then, 330 µl of a 90/10 (v/v) methanol/water mixture with internal standard (ribitol, 0.05 mg/ml) was added. The mixture was shaken for 15 min at 70 °C and then cooled [to room temperature (RT)] and subsequently 230 µl chloroform with standard solution (methylnonadecanoate, 0.25 mg/ml) was added. The mixture was shaken at 37 °C for 5 min; afterwards, 400 µl 18 MΩ water was added and then again shaken at RT for 1 min. The samples were centrifuged for 5 min at 14,000 g and an aliquot of 80 µl of the upper polar phase containing the metabolites was taken and dried in a vacuum concentrator. The oxime reagent was 50 mg of 4-(dimethylamino)pyridine dissolved in 10 ml pyridine with subsequently added 400 mg of methoxyamine hydrochloride. The silylation reagent was a mixture of 1 ml N,O-bis (trimethylsilyl)trifluoroacetamide (BSTFA) and 150 µl retention index solution (containing n-decane, n-hexadecane, n-docosane, n-octacosane, and n-tetratriacontane). For derivatization, 40 µl oxime reagent was added to the dried residue; then, the solution was shaken for 90 min at 30 °C. Subsequently, 80 µl silylation reagent was added, thoroughly mixed for 1 min at 37 °C, and incubated at 37 °C for 30 min. The solution was transferred to a silanized GC-vial and quickly sealed. GC-MS/MS analysis was carried out on an Agilent 7890B gas chromatograph coupled with an Agilent 7000D triple quadrupole mass spectrometer (Agilent, Waldbronn, Germany). The injection volume was 1 µl (splitless). The separation was done on an HP-5MS UI fused silica capillary column (30 m, 0.25 mm I.D., 0.25 mm film thickness), and the injector temperature was set to 250 °C; the carrier gas was He with a flow rate of 0.6 ml min^-1^. The temperature program was 70°C (1 min), followed by an increase of 9 °C min^-1^ to 310 °C (10 min). The transfer line and source temperature were set to 250 °C. The mass selective detector was operated in scan mode with a mass range of m/z 70–600. The identification of metabolites was done *via* the NIST database (2017) and standard substances with respect to retention time and mass spectra. The metabolomics procedure is described in Dethloff et al. (2014).

### 2.6 Lipid profiling

Lipids were extracted from the freeze-dried, pulverized leaf material following the protocol published by Shiva et al. (2018), with minor modifications, reported by Kehelpannala et al. (2020). The freeze-dried maize samples were homogenized by cryomilling (Precellys 24; Bertin Technologies, https://bertin-technologies.com) with 400 µl of 2-propanol containing 0.01 % butylated hydroxytoluene (BHT) for two consecutive 45 s intervals, with a 30 s pause in between, at 6,100 rev min^-1^ and a temperature of −10 °C. Next, the samples were incubated at 75 °C for 15 min while being gently shaken at 1,400 rev min^-1^. Then, they were cooled to room temperature (25 °C), and 1.2 ml of a mixture of chloroform (CHCl_3_)/methanol (MeOH)/water (30/41.5/3.5, v/v/v) was added to each sample. The samples were incubated at 25 °C for 24 h with constant gentle shaking. Finally, the solvent was separated, and the sample was dried in a vacuum concentrator. A quality control sample was prepared by combining 10 µl of each sample extract. The dried lipid extracts were resuspended in 200 µl of butanol (BuOH)/MeOH (1:1) with 10 mM ammonium formate and subjected to LC-MS analysis, as reported by Hu et al. (2008). Extracts were used for untargeted liquid chromatography–mass spectrometry (LC-MS) lipidomics measurement using the protocol of Yu et al. (2018).

### 2.7 Statistical analysis

Lipidomic and metabolomic data (with n = 4–5 replicates, 2 out of 70 samples were lost during preparation) were normalized to library size and log_2_-transformed. For statistical analysis and plotting, the program R was used (Version 4.1.1). We stratified the data with respect to hybrid and timepoint and used the “limma” package (Ritchie et al., 2015) from the Bioconductor project (Huber et al., 2015) to test lipid-wise for an association with treatment. Pairwise comparisons were corrected for multiple testing using the procedure of Benjamini–Hochberg (Benjamini and Hochberg, 1995). The alpha used for the significance of adjusted *p*-values was alpha < 0.05. The t-statistics from the “limma” function was used to generate a pre-ranked lipid “genes” list, which was used to perform a gene set enrichment analysis using the function “cameraPR” (Wu and Smith, 2012) from the “limma” package. The principal component analysis (PCA) was calculated using normalized and log_2_-transformed lipid and metabolite data, which were scaled and centered lipid/metabolite-wise prior to PCA. PCA was calculated using the “prcomp” function from the R “stats” package. For the loadings plot, the loadings of the individual lipids were summarized into a single vector pointing toward the center of gravity of the individual lipids within one lipid class to better illustrate the impact of the whole lipid class.

Log_2_ fold change (logFC) ratios were calculated as follows: the DGDG/MGDG logFC ratio is the ratio of the major thylakoid lipids DGDG to MGDG, calculated as the average logFC(DGDG) – average logFC(MGDG). The plastidial/extraplastidial lipids logFC ratio is the average logFC of plastidial lipids (DGDG, MGDG, SQDG, and PG) relative to the average logFC of extraplastidial phospholipids (PC, PE, PI, and PS). PC/PE is the ratio of the major bilayer (PC) to non-bilayer (PE) lipids in the PM [average logFC(PC) – average logFC(PE)].

For the correlation network, lipid data were stratified by hybrid and timepoint; then, a linear model was fitted using the R function “lm()” for each unique combination of lipids to test for a linear relationship of lipids *via* the treatment group (repeated drought and control or second drought only and control). R² and *p*-values were extracted using the R “summary()” function. Each resulting edge dataset was corrected for multiple testing using the “p.adjust()” function with the “Benjamini–Hochberg” method. The final edge data was generated by filtering each dataset for *p.adj* < 0.05 and R² > 0.9. The nodes (lipids) were arranged in a three-dimensional grid where the z-axis represents the carbon index of the lipids. Lipid vectors with the same carbon index but different number of double bonds were rotated counterclockwise by an angle theta, where theta = 2*π/n+1 (n = maximum number of double bonds per lipid class). The radius of the resulting lipid group cylinder was scaled by the cardinality of the lipid class set relative to the lipid class set with the highest cardinality. The lipid group cylinders were placed inside the planes y = 10 (DGDG, MGDG, SQDG, PG, CL, and HexCer), y = -10 (TG, DG, MG, and sterols), y = -20 [lysophosphatidylcholine (LPC) and lysophosphatidylethanolamine (LPE)], and y = -30 (PC, PE, PI, and PS).

## 3 Results

### 3.1 Photosynthesis, leaf elongation, plant weight, ion leakage, and osmolality

**Figure 2:**
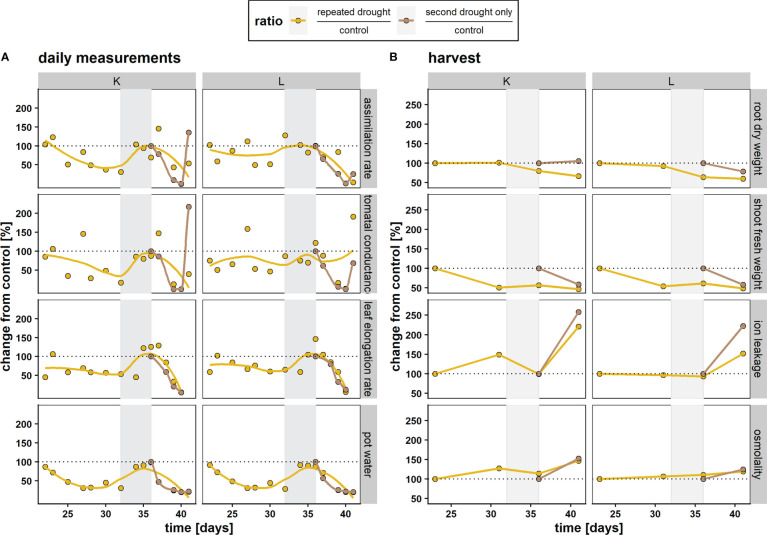
Responses of maize hybrids K and L to repeated drought treatment (yellow) and the second drought–only treatment (brown) relative to well-watered control values in percent (100 %, dotted line). Each datapoint represents the mean of n = 4–5 replicates. Lines were fitted with a lowess function for curve smoothing. Gray area represents the recovery phase. **(A)** Physiological responses of the assimilation rate, stomatal conductance, the leaf elongation rate (A, *gs*, and LER, respectively), and pot water. **(B)** Biomass and stress parameters from destructive sampling. A + B: The first measurement at d 23 of the repeated drought treatment and the first measurement of the second drought–only treatment are imputed values to better illustrate changes because no harvest has taken place before the first drought at d 23 and the second drought–only treatment was at control conditions (= 100 %) after the recovery event.

Both hybrids exhibited a progressive decrease in gas exchange under drought conditions at single drought and repeated drought, respectively (**Figure 2A**). They both showed a return to control conditions during the recovery phase (**Figure 1**, gray area). For K, the response of the assimilation rate and stomatal conductance (**Figure 2A**) was more pronounced (lower in comparison to well-watered control) under the first mild drought, emphasizing higher sensitivity to water deficit. Furthermore, the assimilation rate and stomatal conductance of the repeated drought treatment was less affected at the second drought relative to the second drought–only treatment in both hybrids (**Figure 2A**). The changes in the LER during the experiment were relatively similar in both hybrids and treatments. Both hybrids showed a decline in the LER under repeated drought and an overcompensation toward the end of the recovery phase. When experiencing only the second drought, both hybrids showed a rapid decline in the LER. The root DW and shoot FW responses (**Figure 2B**) were similar in both hybrids, while other stress parameters, such as ion leakage and osmolality, revealed the different sensitivities of the hybrids to mild drought. Ion leakage and osmolality in more drought-sensitive hybrid K were already elevated under first drought, returned to control levels after recovery, and showed rapid elevation under repeated drought in both drought treatments. On the contrary, in the drought-tolerant hybrid L, almost no increase in ion leakage and osmolality was detected after the first drought and only a small increase during the repeated drought (**Figure 2B**). Furthermore, hybrid K shows high ion leakage among both drought treatments after second drought (121 % and 158 % after repeated drought and second drought only, respectively) while hybrid L shows more reduction in ion leakage after repeated drought exposure (52 % and 122 % after repeated drought and second drought only, respectively). Overall, the results were in agreement with previous data (Kränzlein et al., 2022).

### 3.2 Overview of metabolic and lipidomic adaptation

**Figure 3:**
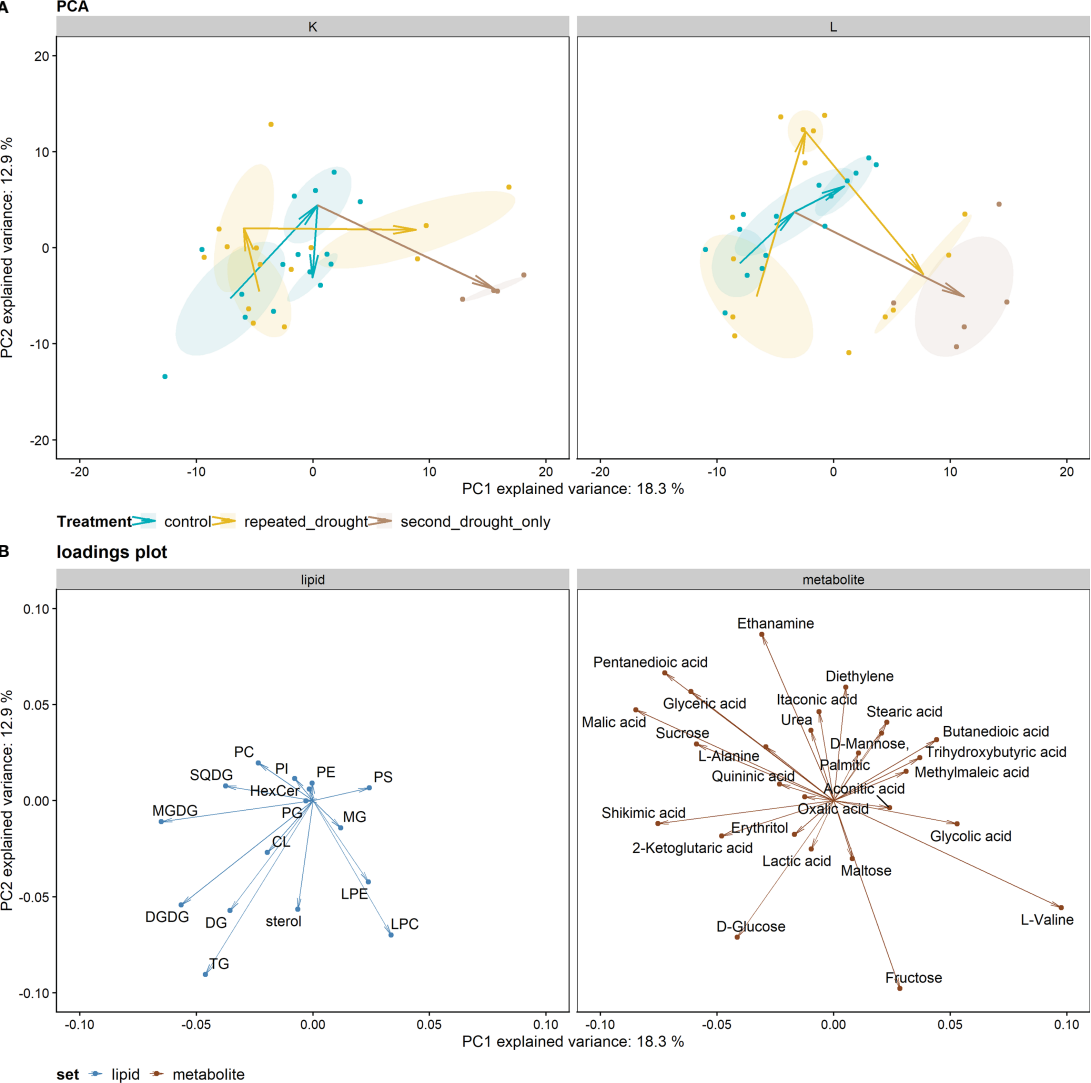
PCA analysis of metabolite and lipid data: **(A)** PCA plot facetted by hybrid. Arrows indicate the progression of time (first drought—recovery—second drought) in each treatment group. Ellipses indicate 95 % confidence intervals of the center of gravity of each subgroup. The second drought–only treatment stems from the control treatment after recovery. **(B)** Plot illustrating the loadings for PCA projection. The arrows that belong to lipid groups point toward the center of gravity of the single lipids within the respective lipid group to better illustrate the impact of the whole lipid group.

For investigating overall adaptation patterns between hybrids across all timepoints and treatments, a PCA was calculated (**Figure 3**). The PCA using metabolite and lipidomic data explained 31 % of total variance *via* the first two principal components, PC1 and PC2, while the treatment was mainly separated by the PC1 (right side: severe drought, left side: control, recovery, and mild drought). The first drought was mild such that the drought treatments were not separated from control in this projection. However, hybrid L showed a more pronounced adaptation after recovery than K. Second drought treatment led to similar changes in both hybrids mainly on the PC1 axis relative to developmental changes in the well-watered controls. The PC1 component separating the treatments correlated most with L-valine, malic acid, shikimic acid, pentanedioic acid, MGDG, glyceric acid, sucrose, DGDG, and glycolic acid (**Figure 3B**).

### 3.3 Metabolite responses

**Figure 4:**
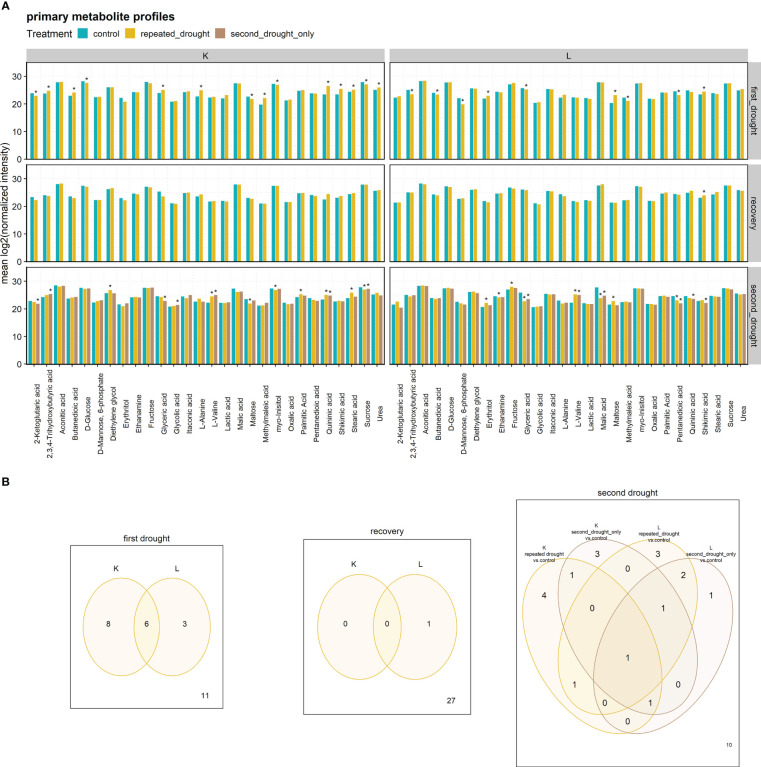
**(A)** Primary metabolite profiles. Stars indicate significant differences to the control of the respective treatment group at the significance level *p.adj* < 0.05. **(B)** Venn diagrams of metabolites significantly altered. Significance was determined as *p.adj* < 0.05.

To investigate the metabolite responses of repeated drought, 27 stress-responsive metabolites were measured (**Figure 4**). Most significant metabolite abundance changes were detected after the first drought priming event (14 in K and 9 in L, with shikimic acid being upregulated in both hybrids). Contrasting changes occurred in butanedioic acid, 2,3,4-trihydroxybutyric acid, glyceric acid, and maltose. After recovery, all metabolite abundances returned to control values, except for shikimic acid in hybrid L. The second drought treatment caused an increase in abundance changes in both hybrids but is lower in numbers than after first drought in the repeated drought treatment (8 in K and 8 in L, with L-valine being upregulated in both hybrids). Contrasting adaptations occurred in maltose. In general, significant fold changes relative to control were increased under drought but mostly returned to control values after rewatering in both hybrids (except for shikimic acid in L).

### 3.4 Lipid profiles and lipid remodeling

**Figure 5:**
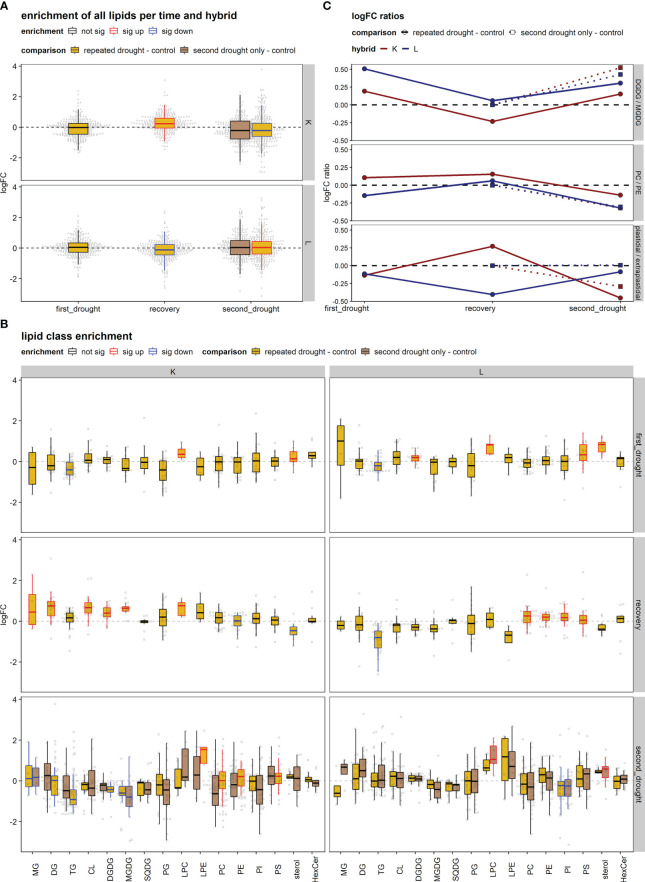
Lipid set enrichment analysis and log_2_ fold change (logFC) ratios. **(A)** Lipid set enrichment of all lipids relative to control per hybrid. **(B)** Lipid set enrichment of lipid classes in the treatment groups per hybrid and experimental stage. The significance of the enrichment is indicated by the color of the boxplot (sig up/down or not sig). **(C)** logFC ratios [i.e., logFC digalactosyl-diacylglycerol (DGDG) – logFC monogalactosyl-diacylglycerol (MGDG)] in the treatment groups per hybrid and experimental stage. The DGDG/MGDG logFC ratio is the ratio of the major thylakoid lipids DGDG (bilayer) to MGDG (non-bilayer). The plastidial/extraplastidial lipids ratio is the average logFC of plastidial membranes [DGDG, MGDG, sulfoquinovosyldiacylglycerol (SQDG), and phosphatidylglycerol (PG)] relative to the average logFC of extraplastidial phospholipids [phosphatidylcholine (PC), phosphatidylethanolamine (PE), phosphatidylinositol (PI), and phosphatidylserine (PS)]. PC/PE is the ratio of the major bilayer (PC) to non-bilayer (PE) lipids.

No significant differences in total lipid sets were detected after the first drought treatments in both hybrids; however, significant differences in abundance from control were detectable after the recovery from drought (**Figure 5A**). The direction of fold changes to control were slightly contrasting in hybrids (0.240 up in K, -0.145 down in L). After the second drought event, a similar contrasting response trend could be observed (-0.190 down in K, 0.073 up in L) in the repeated drought treatment, while the lipid set enrichment was not significant in any direction (up/down) of the second drought–only treatment.

We then investigated patterns of lipid remodeling within lipid class sets (**Figure 5B**). After the first drought, hybrid K showed the downregulation of TG lipids and the upregulation of LPC and sterol lipids. L showed higher LPC, PS, and sterol lipids and lower TG [and increased DGDG likely through MGDG to DGDG conversion leading to the observed increase in DGDG/MGDG ratio (**Figure 5C**)]. After recovery, hybrid K showed significant increases in the lipid classes of MG, DG, LPC, CL, DGDG, and MGDG and significant reduction in PE and sterol lipids. In contrast, L showed significant increase in PM phospholipids and reduction in TG. After the second drought in K, significant increases occurred in the lipid classes of LPE, PC, PE, and PS and decreases in MG DG, TG, DGDG, and MGDG upon the repeated drought treatment, while MG and MGDG decreased in the second drought–only treatment. In contrast in L, only LPC and sterol were increased in the second drought–only treatment, while PI decreased in both drought treatments.

The plastidial/extraplastidial lipids logFC ratio (**Figure 5C**) showed a tendency to decrease after first drought in both hybrids. After recovery, the plastidial/extraplastidial lipids logFC ratio was elevated in hybrid K, due to the increased synthesis of chloroplast lipids, while extraplastidial phospholipids remained at the control level except for PI, which decreased slightly (**Figure 5B**, K recovery). In contrast in hybrid L, the plastidial/extraplastidial lipids logFC ratio was further decreased after recovery, as chloroplast lipids decreased or remained at control levels, while the synthesis of phospholipids was promoted (**Figure 5B**, K recovery). After the second drought, the plastidial/extraplastidial lipids logFC ratio was decreased in both hybrids in the repeated drought treatment but to a higher extent in K. Furthermore, the second drought–only treatment displayed less change in the logFC ratio in both hybrids.

The DGDG/MGDG logFC ratios (**Figure 5C**) increased in both hybrids under first and second drought but to a higher extent in L. After recovery, the DGDG/MGDG logFC ratio returned to control values in tolerant hybrid L, while hybrid K produced more MGDG relative to DGDG, leading to a lower logFC ratio after recovery. After second drought, logFC ratios increased in all treatments except for repeated drought in K, indicating reduced capability to increase DGDG/MGDG ratio under repeated drought.

The PC/PE ratio remained almost constant throughout the experiment, there was a slight tendency of an increased PC/PE ratio after recovery in both hybrids, and a slight reduction under drought conditions (except for hybrid K after first drought) could be observed (**Figure 5C**).

**Figure 6:**
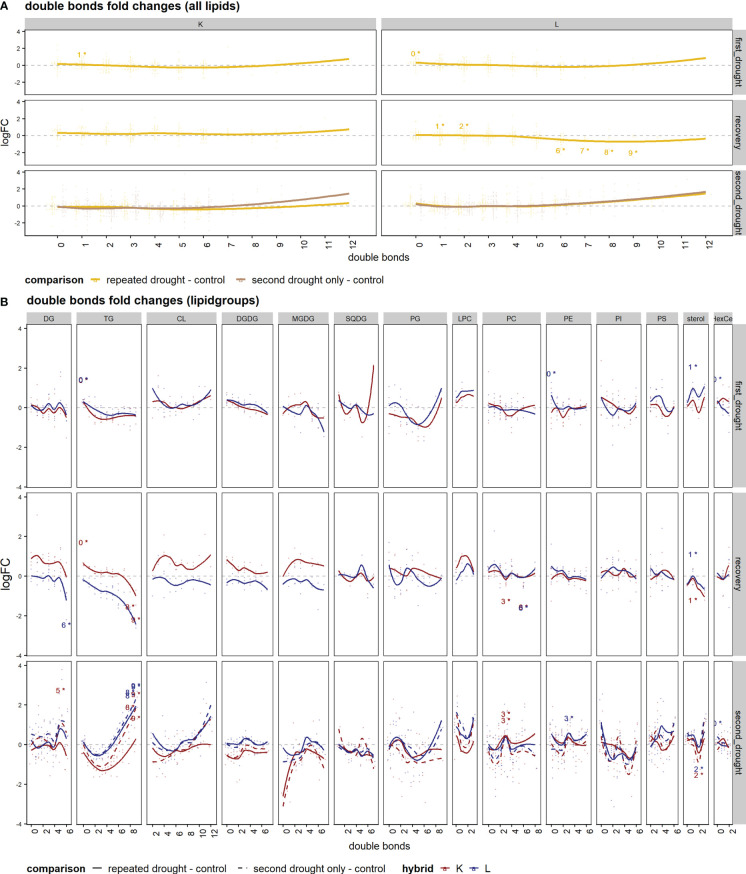
Set enrichment of double bond fold changes. **(A, B)** The average logFC of saturation patterns of lipids represented with numbers of double bonds in comparison of drought treatments and the well-watered control. **(A)** The mean of all lipid species. **(B)** Single-lipid species of both hybrids; lines were fitted with a lowess function. Text annotations indicate the significance of the respective set enrichment; the number refers to the lipid set that is significantly enriched (the number of double bonds) and the star "*" indicates a significant difference at *p.adj* < 0.05.

Next, we wanted to understand the adaption of membrane fluidity under repeated drought. We investigated the average logFC of fatty acid saturation patterns of all lipids represented as the number of double bonds for drought treatments and the control (**Figure 6A**) and of single-lipid species (**Figure 6B**), respectively. The levels of fatty acid saturation of all lipids at first drought were similar in both hybrids: an induction of high and low double-bond lipids and a slight reduction of three to eight double-bond lipids was observed (**Figure 6A**). This change was significant in the case of low double bonds in both hybrids (**Figure 6A**). The main difference between the hybrids became apparent after the recovery phase, where hybrid K appeared to adapt by a tendency to especially increase highly unsaturated lipids (change is not significant). On the contrary in L, medium double-bond lipids (six to nine double bonds) were significantly decreased, and one to two double bonds were increased after recovery. After the second drought, the 7–12 double-bond lipids showed a tendency of increase in both hybrids. An exception from this trend was the second drought–only treatment in K, which showed no tendency of increase in the higher double bonds relative to control, and this could indicate a lesser potential to adjust membrane fluidity in this hybrid with previous mild drought exposure. More differences in responses became visible in the lipid class analysis (**Figure 6B**): after first drought, patterns of double bond responses were similar in hybrids except for sterols, which displayed higher fold change in L (**Figure 6B**, first drought). After recovery, genotypic differences arose within the lipid groups of DG, TG, CL, DGDG, MGDG, and LPC, which show higher logFC patterns in K (**Figure 6B**, recovery). After the second drought, the patterns of a subset of the same lipid classes (TG, CL, DGDG, MGDG, and LPC) as well as PS showed higher logFC in L than in K (**Figure 4B**, second drought). Furthermore, the second drought–only treatment in K maintained higher logFC than repeated drought in the classes of DG, TG, CL, and LPC, indicating that previous drought and recovery modulated the response to second drought in K, leading to higher decline in some lipid species, especially in TG’s with four to nine double bonds.

**Figure 7:**
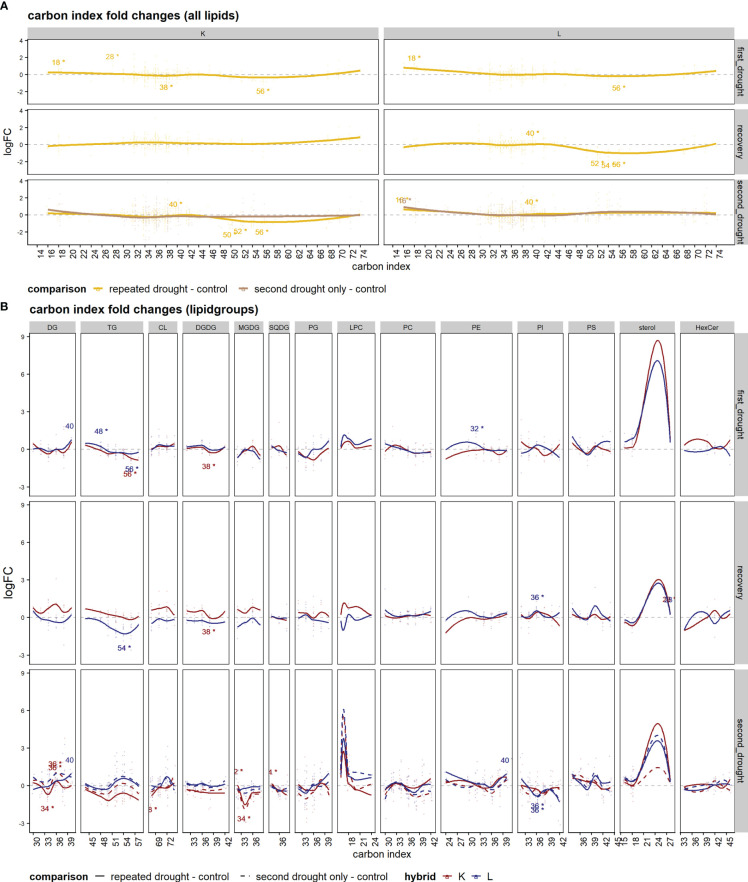
Set enrichment of C-index fold changes. **(A, B)** Average logFC of the lipid carbon index of the comparison of drought treatments and the control: **(A)** the mean of all lipid species and **(B)** single-lipid species of both hybrids. Lines were fitted with a lowess function. Text annotations indicate the significance of the respective set enrichment; the number refers to the lipid set that is significantly enriched (C-index) and the star "*" indicates a significant difference at *p.adj* < 0.05.

When considering the lipid carbon atom index (**Figure 7**), the overall lipid response patterns over time resembled what was also detected for double bonds, a similar response after first drought in both hybrids with increasing small lipids, while medium-sized lipids remained around control levels, and a slight decrease in bigger lipids between 46 and 70 C-atoms was observed (**Figure 7A**, first drought). At recovery, hybrids diverged in responses as hybrid L showed the downregulation of lipids with a C-index of 46–70, while in K, C-index patterns returned to the control level or were slightly upregulated in the large lipid sets > 64 C-atoms (**Figure 7A**, recovery). The second drought exposure led to a decline in lipids with the C-index between 46 and 74 in the repeated drought treatment in K, whereas other lipids were slightly upregulated or remain at control levels in the second drought–only treatment in K and in both treatments in L (**Figure 7A**, second drought). The lipid class analysis of C-indices (**Figure 7**) was in agreement with the double-bond lipid class analysis, with most differences aroused after the recovery phase in the lipid classes of DG, TG, CL, DGDG, MGDG, and LPC. The same classes were also affected after second drought between hybrids and with respect to the treatments.

## 4 Discussion

### 4.1 Maize hybrid L is more tolerant than hybrid K to repeated drought treatments

The two maize hybrids L and K display similar reductions in growth parameters, such as relative fresh weight maintenance in response to repeated drought (**Figure 2**). However, differences in gas exchange, ion leakage, and osmolarity were observed. Those differences suggest that hybrid L maintains a drought stress memory after a drought stimulus that enables it to maintain growth better when exposed to a second drought treatment. It appears that hybrid K is more sensitive to drought stress because it does not retain such a stress memory. This is in line with results obtained in a previous study investigating repeated drought in the same maize genotypes (Kränzlein et al., 2022).

### 4.2 Contrasting lipid remodeling is responsible for different genotypic responses to second drought

To investigate the above-mentioned differences of both hybrids in ion leakage, we hypothesized that this discrepancy might be visible in lipid remodeling patterns since cell membranes are early targets of oxidative stress. Comparing total lipid set enrichments, there is a tendency of increased total lipid abundance under drought in the tolerant hybrid L (**Figure 3A**), possibly indicating tolerance, as a decreased lipid content after drought relative to well-watered control is more common (Liu et al., 2019). In line with this tendency, sensitive hybrid K shows decreased lipid contents under severe drought (**Figure 5A**). The first drought was relatively mild, such that tolerant hybrid L showed less physiological responses, while K showed higher ion leakage, osmolality, and the impairment of gas exchange and photosynthesis (**Figure 2**). However, for the individual lipids (**Figures S1–S5**), hybrid L had a higher number of altered lipids than K after second drought in the repeated drought treatment (**Figure S6**). In line with this, hybrid L also showed more correlations not only between lipids after first drought in general but also between different lipid classes (**Figure S7**). A greater number of significantly (*p* < 0.05) altered abundances in hybrid L were also observed in a proteomic study of the same experiment (Schulze et al., 2021). It was hypothesized that as hybrid L had a higher abundance of ribosomal proteins even under well-watered conditions, it could enable a rapid replacement of proteins and a broader response, which might be an important contribution to the robustness of hybrid L under drought conditions. The same observation could be made in this lipidomic analysis, reflecting a broader response than hybrid K but lower in magnitude. Furthermore, the unsaturation of the lipidome was increased after mild drought in both hybrids (**Figure 6A**). This suggests that the lipid remodeling is elicited before cellular damage may occur (low ion leakage in L after first drought), which has been reported in some plant species (Sahsah et al., 1998) and might be another important reason for the drought tolerance of L. Interestingly, the first drought event also altered the lipidome of L considerably after recovery, both in terms of singular lipid changes and changes in the unsaturation of the lipidome, which was reduced (**Figures 6A, B**, recovery). This points toward a memory formation or continuous adaptation occurring in L.

Additionally, differences in lipid remodeling between hybrids were observed in the predominant changes on thylakoid lipids in hybrid K, while, in L, most changes occur on phospholipids and sterols, which is reflected in the plastidial/extraplastidial lipids logFC ratio (**Figure 5C**). In line with this, the correlation network (**Figures S7–S10**) also indicated a prominent role of sterols and phospholipids in L, especially after not only first drought but also second drought in terms of correlations between extraplastidial phospholipids (**Figure S7**, **Figure S9**). Conversely, extraplastidial phospholipids in hybrid K were less interconnected with each other after second drought but showed a high number of correlations to TG and DG, which, in turn, were correlated with galactolipids, forming connected components. This could indicate a high lipid turnover rate *via* TG and DG in K (**Figure S9**). It seems possible that a rigidification of the PM occurs in tolerant hybrid L at the expense of photosynthetic processes, but this rigidification could be advantageous in upcoming drought. It can be observed that the chloroplast lipids were less damaged relative to the PM lipids in the repeated drought treatment in L because the plastidial/extraplastidial lipids logFC ratio increased relative to the logFC ratio after recovery in this treatment (**Figure 5C**). Conversely, the ratio of the major bilayer PM phospholipid PC and the major non-bilayer phospholipid PE remained almost constant throughout the experiment, showing only a slight decrease after second drought in both hybrids (**Figure 5C**). A stable PC/PE ratio was also seen in a recent study on sorghum during salt stress (Ge et al., 2022). Furthermore, LPC and LPE are known stress signaling molecules in low concentrations but can be lipotoxic in high concentrations (Liu et al., 2019). In hybrid K, LPC and/or LPE are high in relative abundance, especially LPE under severe drought in the repeated drought treatment (**Figure 5C**), which could hint for lipotoxic processes. In hybrid L, LPC is only significantly increased after first drought and after second drought in the second drought–only treatment, which suggests a better control of lipotoxic processes in the repeated drought treatment (**Figure 5C**).

The adaptation strategies based on lipid remodeling are inherently different in both hybrids, showing contrasting patterns of lipid adaptation, especially during the recovery phase. These differences in lipid patterns may reflect their tolerance strategies, where hybrid L achieves a more effective response toward repeated drought, while hybrid K shows a strong recovery response, which appears to be less effective toward an upcoming drought in terms of effective lipid remodeling.

#### 4.2.1 Ability to adjust digalactosyl-diacylglycerol/monogalactosyl-diacylglycerol ratio and fatty acid unsaturation under drought contributes to tolerance

The DGDG/MGDG ratio is a suitable indicator for thylakoid lipid remodeling under drought and other abiotic stresses (Gigon et al., 2004; Gasulla et al., 2013). The elevated DGDG/MGDG ratio under dehydration arises mainly by a reduction in MGDG, an increase of DGDG, and conversion from MGDG to DGDG (Gigon et al., 2004; Chen et al., 2018; Du et al., 2018; Liu et al., 2019). This is thought to stabilize the thylakoid bilayer structure, preventing the accumulation of ROS and photodamage based on the observation that mutants lacking MGDG conversion to DGDG and DG/TG under drought in Chlamydomonas lead to a grana hyperstacking phenotype, and, since photosystem II-complexes are mostly located in the grana, while photosystem I-complexes are mainly located in the stroma, this can lead to a higher PSII/PSI ratio (Du et al., 2018). When growth is reduced by drought, the photosynthetic apparatus and photosynthetic membranes may also be reduced to prevent the production of ROS by excess light-harvesting activity (Du et al., 2018). It is discussed that the DGDG/MGDG ratio adjustment is an adaptation strategy rather than an indicator for stress-associated damage, and the ability to increase the DGDG/MGDG ratio under abiotic stress contributes to higher tolerance (Chen et al., 2018).

In this experiment, the different adjustments of the DGDG/MGDG logFC ratio between contrasting hybrids agrees with this hypothesis, as tolerant hybrid L reaches a higher ratio during both stress periods than sensitive hybrid K under repeated drought (**Figure 5B**). Furthermore, the second drought–only treatment in K achieved a much higher logFC ratio than repeated drought treatment, indicating that the previous drought and rewatering cycle hampered the ability of K to adjust the DGDG/MGDG ratio under drought. The reason for this could be the strong upregulation of both MGDG and DGDG after recovery (but to a higher-degree MGDG, which leads to a lower ratio in K, **Figure 5B**), and those lipid species are most susceptible to drought stress (Sahsah et al., 1998; Matos et al., 2002). It can be hypothesized that K has lost the ability to efficiently convert MGDG to DGDG under drought conditions, such that the excess in MGDG could not be converted, but was rather degraded through oxidative processes, similar as observed in Chlamydomonas mutants lacking MGDG conversion (Du et al., 2018). In line with this, as observed in a proteomic study of the experiment (Schulze et al., 2021), it was measured that PSII complexes were upregulated in K after repeated drought, which could have led to an increased PSII/PSI ratio in K, thus leading to the production of ROS. However, having high amounts of MGDG and DGDG might contribute to better growth and photosystem efficiency under well-watered conditions or at poststress. This hypothesis is supported by the observation that the assimilation rate and stomatal conductance in K are more sensitive under drought (**Figure 2**) but recover quickly upon rewatering, leading to even higher rates of gas exchange up to 1 d after the beginning of second drought. In hybrid K, the fatty acid unsaturation adaptability is higher in the second drought–only treatment than in repeated drought treatment (**Figure 6A**, repeated vs. second drought only). Moreover, the fatty acid unsaturation levels were already increased before the onset of the second drought in K (**Figure 6A**), suggesting that the increased initial unsaturation could not be maintained under severe drought. Conversely, in hybrid L, unsaturation levels were reduced after recovery but could be increased after the repeated drought (**Figure 6A**, hybrid L recovery vs. second drought, repeated drought treatment, and highly unsaturated lipids). A higher unsaturation of lipids is thought to increase the resistance to various abiotic stresses such as drought (Monteiro de Paula et al., 1993; Gigon et al., 2004; Zhang et al., 2005; Zheng et al., 2021) or low temperature (Vijayan and Routaboul, 1997; Cheong et al., 2019). Therefore, it has been suggested that increased levels of unsaturation of lipids under stress is an important trait of stress-tolerant plants (Repellin et al., 1997; Gigon et al., 2004). We hypothesize that the ability to increase or maintain unsaturation under drought contributes to tolerance, and this ability was hampered in hybrid K after second drought in the repeated drought treatment.

#### 4.2.2 Importance of the recovery state for different adaptation strategies

The recovery phase is a crucial phase where plants either keep an adapted state or reset the information obtained during drought priming (Crisp et al., 2016). The changes made during the recovery phase have a potentially high impact on the fitness of the plant toward future stress events (Hilker & Schmülling, 2019). In our experiment, significant changes with contrasting dynamics were made during the recovery period. In hybrid K, most adaptations of lipid species like MGDG, DGDG, or CL occur at or after recovery and might lead to a quicker recovery of growth rates (**Figure 2**, leaf elongation rate in K). However, the remodeling was ineffective to cope with upcoming drought compared with hybrid L and compared with second drought–only treatment. It is intriguing that DG is accumulated in hybrid K after recovery, but not under single drought before recovery, since DG is known to be produced during abiotic stress and declines thereafter (Gasulla et al., 2013). However, DG cannot be sufficiently produced after the second drought in the repeated drought treatment in K compared with the second drought treatment (and compared with the drought treatments in hybrid L). Additionally, as suggested above, it seems that hybrid K could not convert the excess MGDG produced after recovery into DGDG and DG/TG under repeated drought, leading to a lower DGDG/MGDG logFC ratio and therefore an impairment in adjusting chloroplast ultrastructure under drought conditions. This would cause a hyperstacking of grana which in turn might lead to production of ROS through excess light-harvesting activity (Du et al., 2018), causing the inability for a directed lipid remodeling, as it occurs in hybrid L. Moreover, CL is being produced along with MGDG and DGDG during recovery in hybrid K (**Figure 5C**). The drought priming could have elicited mitochondrial and chloroplast proliferation, as they synergize *via* a mitochondrial and chloroplast crosstalk (Zottini, 2013), which together might lead to the strong recovery response seen in this hybrid. In general, the lipid remodeling during or after the recovery phase is crucial for the explanation of the divergence of stress adaptation strategies between the hybrids. In contrast, both hybrids show a return to control conditions after recovery in terms of metabolites, except for shikimic acid in hybrid L, which remains high during the first drought until the end of the recovery period (**Figure 4**). This might indicate an increased synthesis of phenolic compounds and antioxidants in L persisting until after 4 d of recovery. It can be stated that the overcompensative lipid remodeling during the recovery phase in hybrid K might contribute to an impairment of lipid remodeling in the subsequent drought, but this overcompensation of lipid synthesis could be beneficial for restarting growth.

### 4.3 Conclusion

The overall responses of two contrasting maize hybrids K and L to drought stress were analyzed with a focus on lipid remodeling. In contrast to growth, which was similarly impaired in both hybrids, ion leakage and gas exchange were mainly impaired under drought in hybrid K. We provide evidence that lipid remodeling, which is inherently different in the two hybrids, presumably plays a central role in drought adaptation. The recovery phase is the most important phase for lipidomic adaptation, and changes during the recovery phase impact on fitness toward future stress. In the case of recovery-oriented hybrid K, the ability for effective lipid remodeling was reduced after recovery in the repeated drought treatment in comparison to the second drought–only treatment. More specifically, the ability to increase the levels of fatty acid unsaturation and the DGDG/MGDG logFC ratio under drought are important traits, and these were hampered in the sensitive hybrid K after the first drought-rewatering cycle. On the other hand, tolerant hybrid L displayed more focus on phospholipid remodeling while efficient adjustments of DGDG/MGDG logFC ratio and unsaturation could be retained after repeated drought.

## Data availability statement

The raw data supporting the conclusions of this article will be made available by the authors, without undue reservation.

## Author contributions

MK planned and conducted the experiment, did data analysis, interpreted results, and wrote the manuscript. SS analyzed data, interpreted results, and wrote the manuscript. C-MG planned the experiment and interpreted results. WS analyzed data, interpreted results, and wrote the manuscript. MA instructed the statistical analysis and interpreted results. HH instructed the metabolite measurement and interpreted results. UR instructed the lipidomics measurement and interpreted results. CZ planned the experiment, interpreted results, and wrote the manuscript. All authors contributed to the article and approved the submitted version.

## Supplementary material

Supplementary Figure 1 Raw intensities of lipid profiles for lipid classes MG, TG, DG facetted by hybrid and timepoint. The x axis labels represent the C-index and double bonds respectively. Stars indicate significant differences between the respective treatment group and the control at *p.adj* < 0.05 (of the library size normalized and log_2_ transformed data)

Supplementary Figure 2 Raw intensities of lipid profiles for lipid classes PC, PE facetted by hybrid and timepoint. The x axis labels represent the C-index and double bonds respectively. Stars indicate significant differences between the respective treatment group and the control at *p.adj* < 0.05 (of the library size normalized and log_2_ transformed data)

Supplementary Figure 3 Raw intensities of lipid profiles for lipid classes PG, PI, PS facetted by hybrid and timepoint. The x axis labels represent the C-index and double bonds respectively. Stars indicate significant differences between the respective treatment group and the control at *p.adj* < 0.05 (of the library size normalized and log_2_ transformed data)

Supplementary Figure 4 Raw intensities of lipid profiles for lipid classes DGDG, MGDG, SQDG facetted by hybrid and timepoint. The x axis labels represent the C-index and double bonds respectively. Stars indicate significant differences between the respective treatment group and the control at *p.adj* < 0.05 (of the library size normalized and log_2_ transformed data).

Supplementary Figure 5 Raw intensities of lipid profiles for lipid classes CL, HexCer, LPC, LPE, sterol facetted by hybrid and timepoint. The x axis labels represent the C-index and double bonds respectively. Stars indicate significant differences between the respective treatment group and the control at *p.adj* < 0.05 (of the library size normalized and log_2_ transformed data).

Supplementary Figure 6 Venn diagrams for lipid profile statistics at *p.adj* < 0.05.

Supplementary Figure 7 Correlation network of lipids in hybrid LK (A) and hybrid KL (B) after the first drought event. The orange edges represent correlations (*p.adj* < 0.05, R² > 0.9) between different lipid classes. Correlations between lipids of the same class share the same edge and node colors. C-indices and double bonds are annotated for 0, 5 and 10 double bonds.

Supplementary Figure 8 Correlation network of lipids in hybrid LK (A) and hybrid KL (B) after recovery. The orange edges represent correlations (*p.adj* < 0.05, R² > 0.9) between different lipid classes. Correlations between lipids of the same class share the same edge and node colors. C-indices and double bonds are annotated for 0, 5 and 10 double bonds.

Supplementary Figure 9 Correlation network of lipids in hybrid LK (A) and hybrid KL (B) after second drought in the repeated drought treatments. The orange edges represent correlations (*p.adj* < 0.05, R² > 0.9) between different lipid classes. Correlations between lipids of the same class share the same edge and node colors. C-indices and double bonds are annotated for 0, 5 and 10 double bonds.

Supplementary Figure 10 Correlation network of lipids in hybrid K L (A) and hybrid L K (B) after second drought in the second drought only treatments. The orange edges represent correlations (*p.adj* < 0.05, R² > 0.9) between different lipid classes. Correlations between lipids of the same class share the same edge and node colors. C-indices and double bonds are annotated for 0, 5 and 10 double bonds.

## Glossary

**Table:**

A	assimilation rate
CL	cardiolipin
d	day
DG	diacylglycerol
DGDG	digalactosyldiacylglycerol
DW	dry weight
FA	fatty acid
FW	fresh weight
Glc	Glc-sterol lipids (e.g.Glc-sitosterol)
*gs*	stomatal conductance
h	hour
Hex	hexosylceramide (sphingolipid)
K	maize hybrid ‘KWS-stabil’
L	maize hybrid ‘LG30222’
LC-MS	liquid chromatography–mass spectrometry
LER	leaf elongation rate
logFC	log_2_ fold change
LPC	lysophosphatidylcholine
LPE	lysophosphatidylethanolamine
MG	monoacylglycerol
MGDG	monogalactosyldiacylglycerol
PC	phosphatidylcholine
PCA	principal component analysis
PE	phosphatidylethanolamine
PG	phosphatidylglycerol
PI	phosphatidylinositol
PM	plasma membrane
PS	phosphatidylserine
ROS	reactive oxygen species
SQDG	sulfoquinovosyldiacylglycerol
TG	triacylglycerol
WHC	water-holding capacity
