# Supplementary figures and images for: Lipid remodeling of contrasting maize (Zea mays L.) hybrids under repeated drought
Source: Front Plant Sci. 2023 May 10;14:1050079. doi: 10.3389/fpls.2023.1050079 (PMC10206266; doi:10.3389/fpls.2023.1050079)

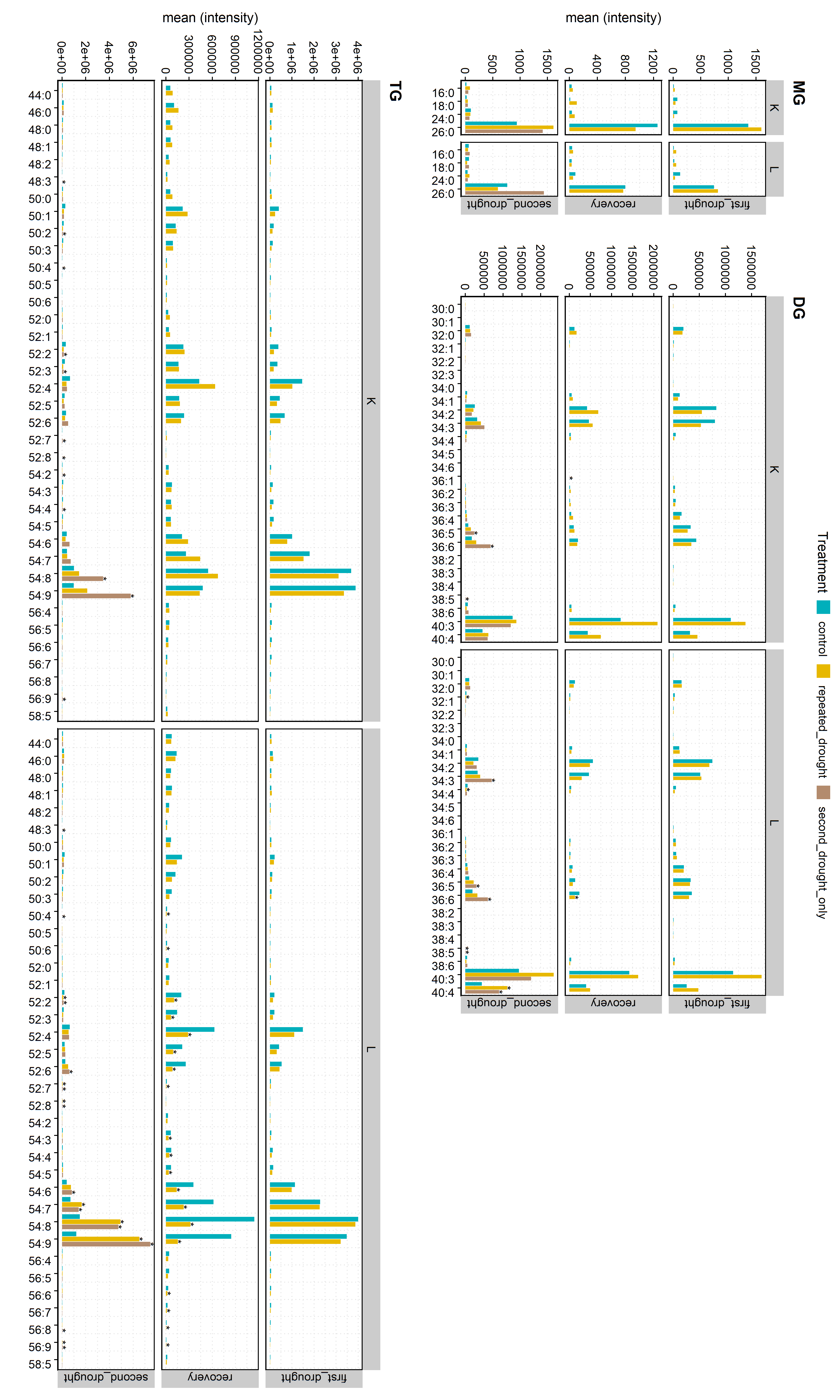

Supplement: Supplementary Figure 1 — Raw intensities of lipid profiles for lipid classes MG, TG, DG facetted by hybrid and timepoint. The x axis labels represent the C-index and double bonds respectively. Stars indicate significant differences between the respective treatment group and the control at p.adj < 0.05 (of the library size normalized and log2 transformed data) [file Image_1.tiff]

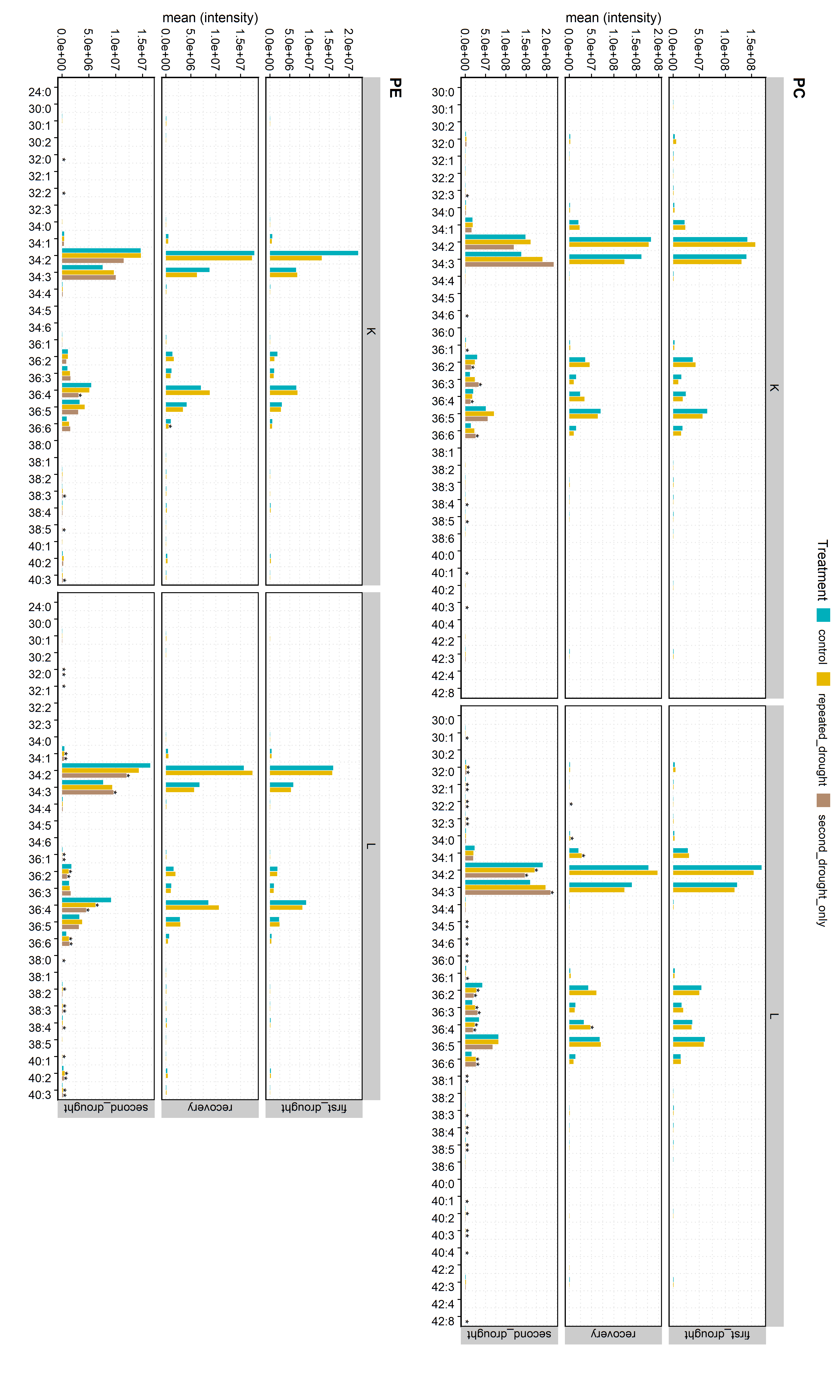

Supplement: Supplementary Figure 2 — Raw intensities of lipid profiles for lipid classes PC, PE facetted by hybrid and timepoint. The x axis labels represent the C-index and double bonds respectively. Stars indicate significant differences between the respective treatment group and the control at p.adj < 0.05 (of the library size normalized and log2 transformed data) [file Image_2.tiff]

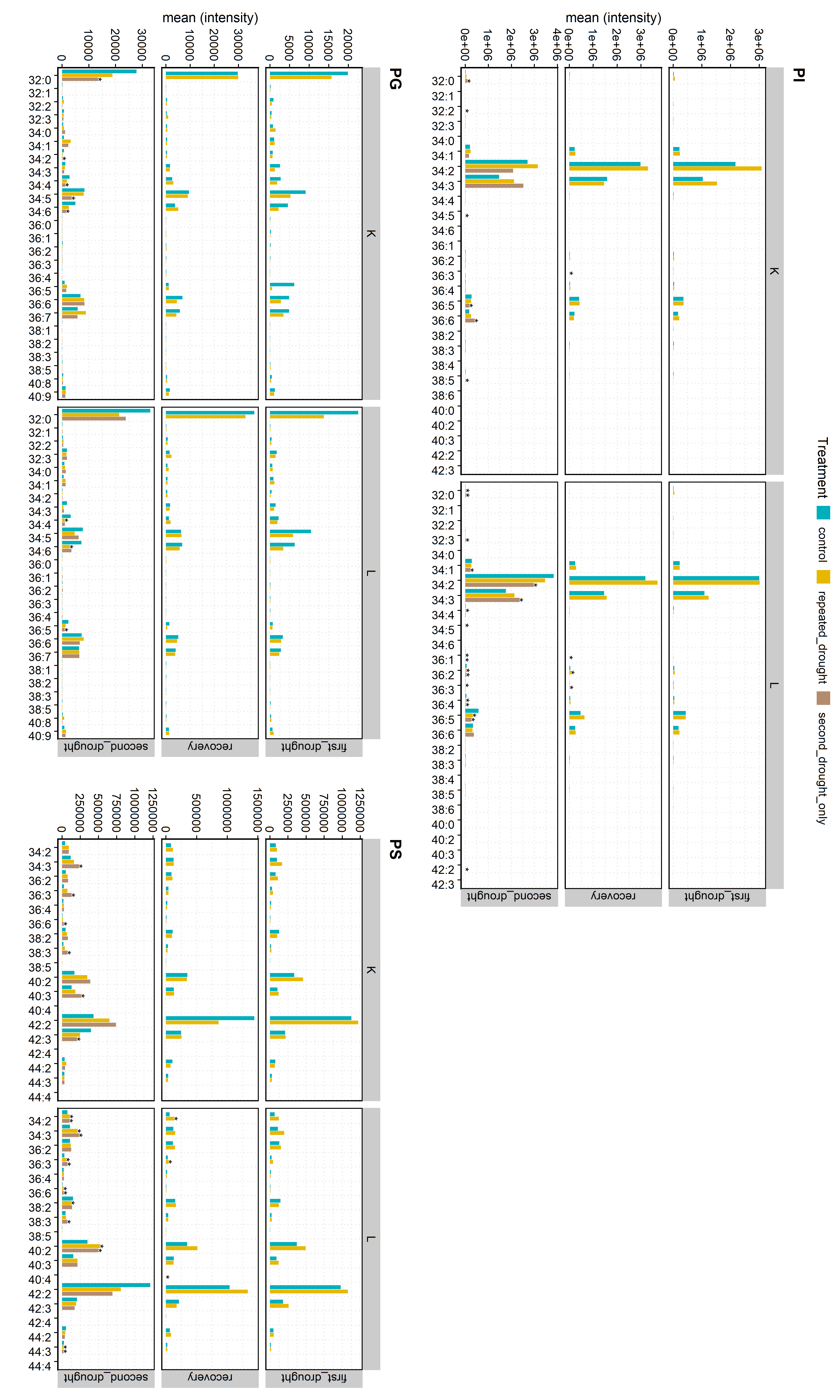

Supplement: Supplementary Figure 3 — Raw intensities of lipid profiles for lipid classes PG, PI, PS facetted by hybrid and timepoint. The x axis labels represent the C-index and double bonds respectively. Stars indicate significant differences between the respective treatment group and the control at p.adj < 0.05 (of the library size normalized and log2 transformed data) [file Image_3.tiff]

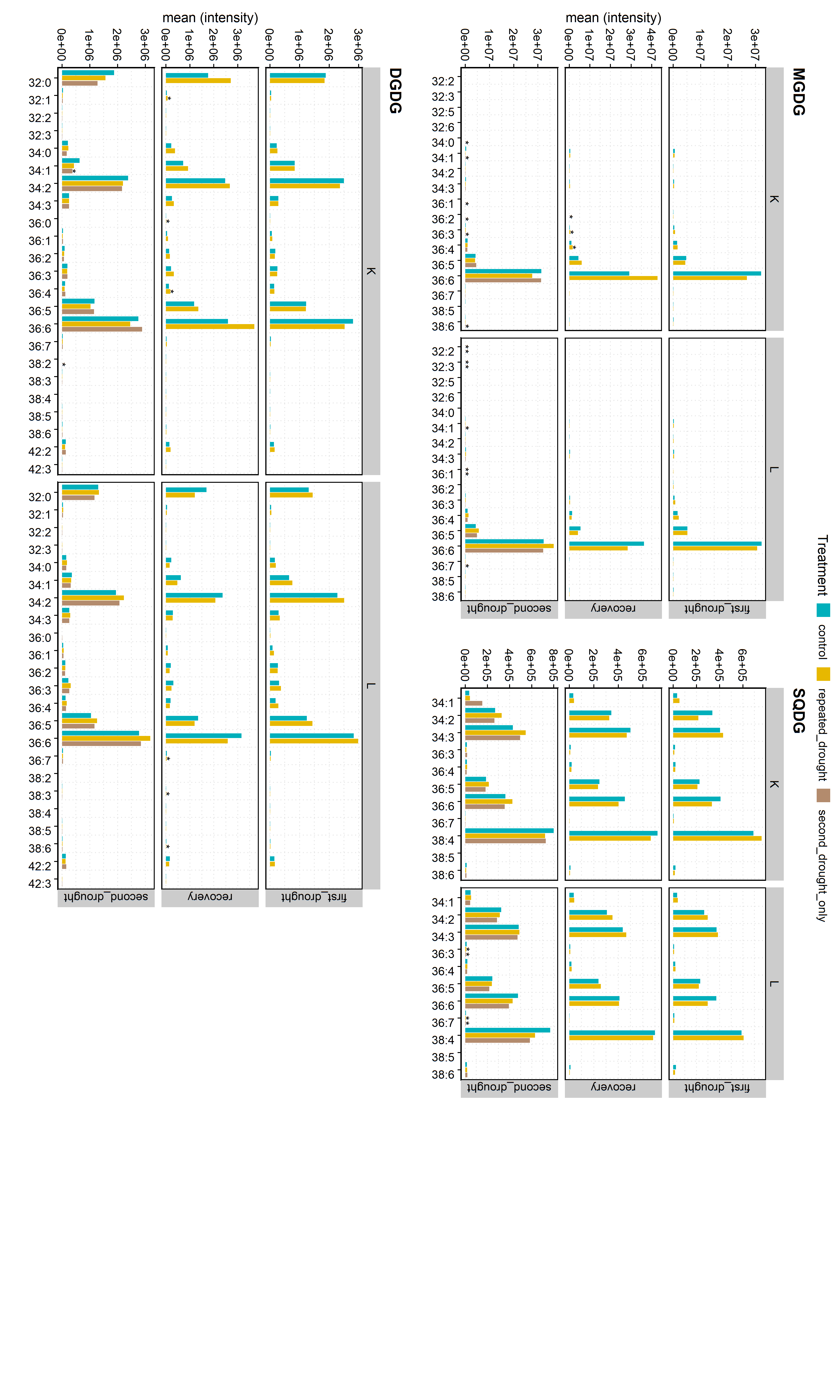

Supplement: Supplementary Figure 4 — Raw intensities of lipid profiles for lipid classes DGDG, MGDG, SQDG facetted by hybrid and timepoint. The x axis labels represent the C-index and double bonds respectively. Stars indicate significant differences between the respective treatment group and the control at p.adj < 0.05 (of the library size normalized and log2 transformed data). [file Image_4.tiff]

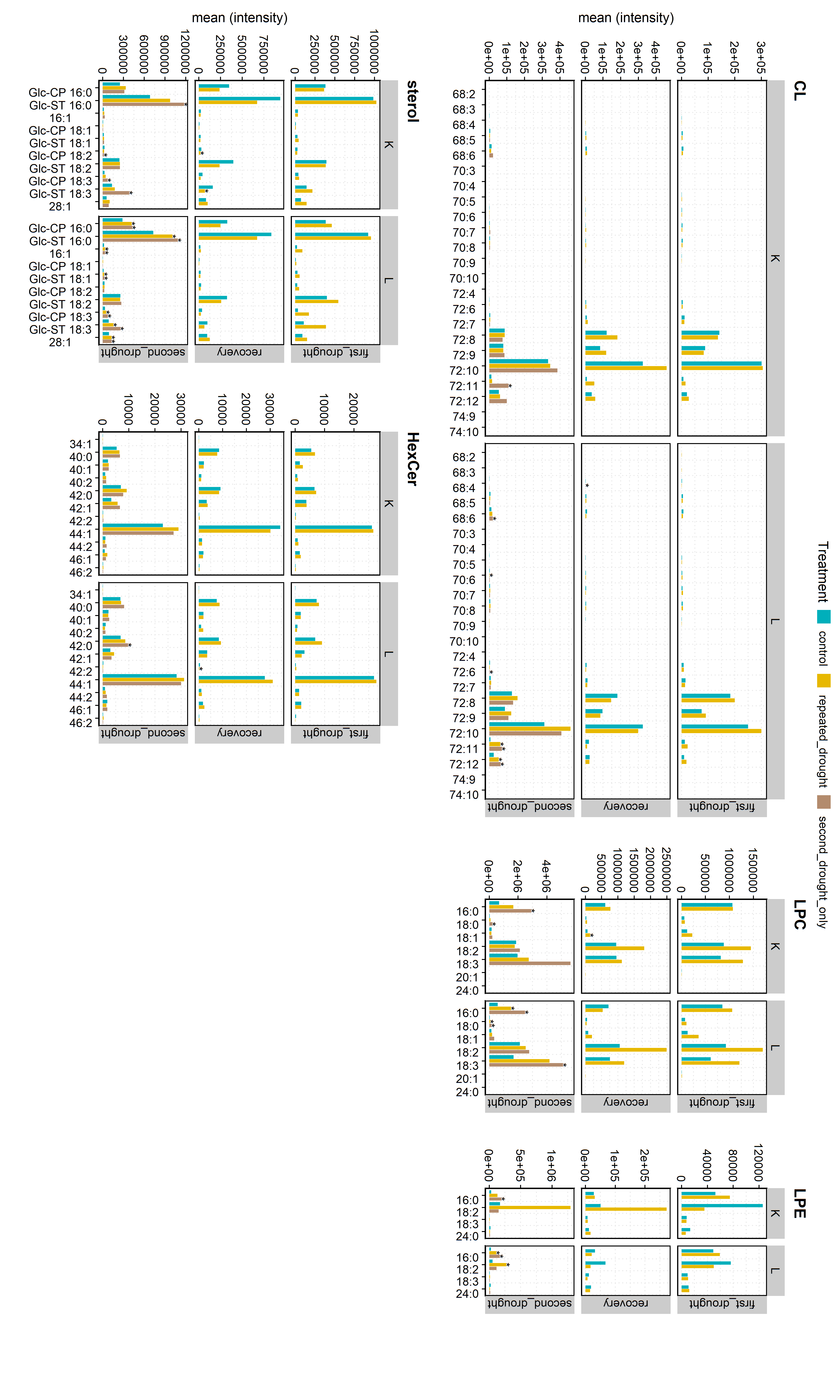

Supplement: Supplementary Figure 5 — Raw intensities of lipid profiles for lipid classes CL, HexCer, LPC, LPE, sterol facetted by hybrid and timepoint. The x axis labels represent the C-index and double bonds respectively. Stars indicate significant differences between the respective treatment group and the control at p.adj < 0.05 (of the library size normalized and log2 transformed data). [file Image_5.tiff]

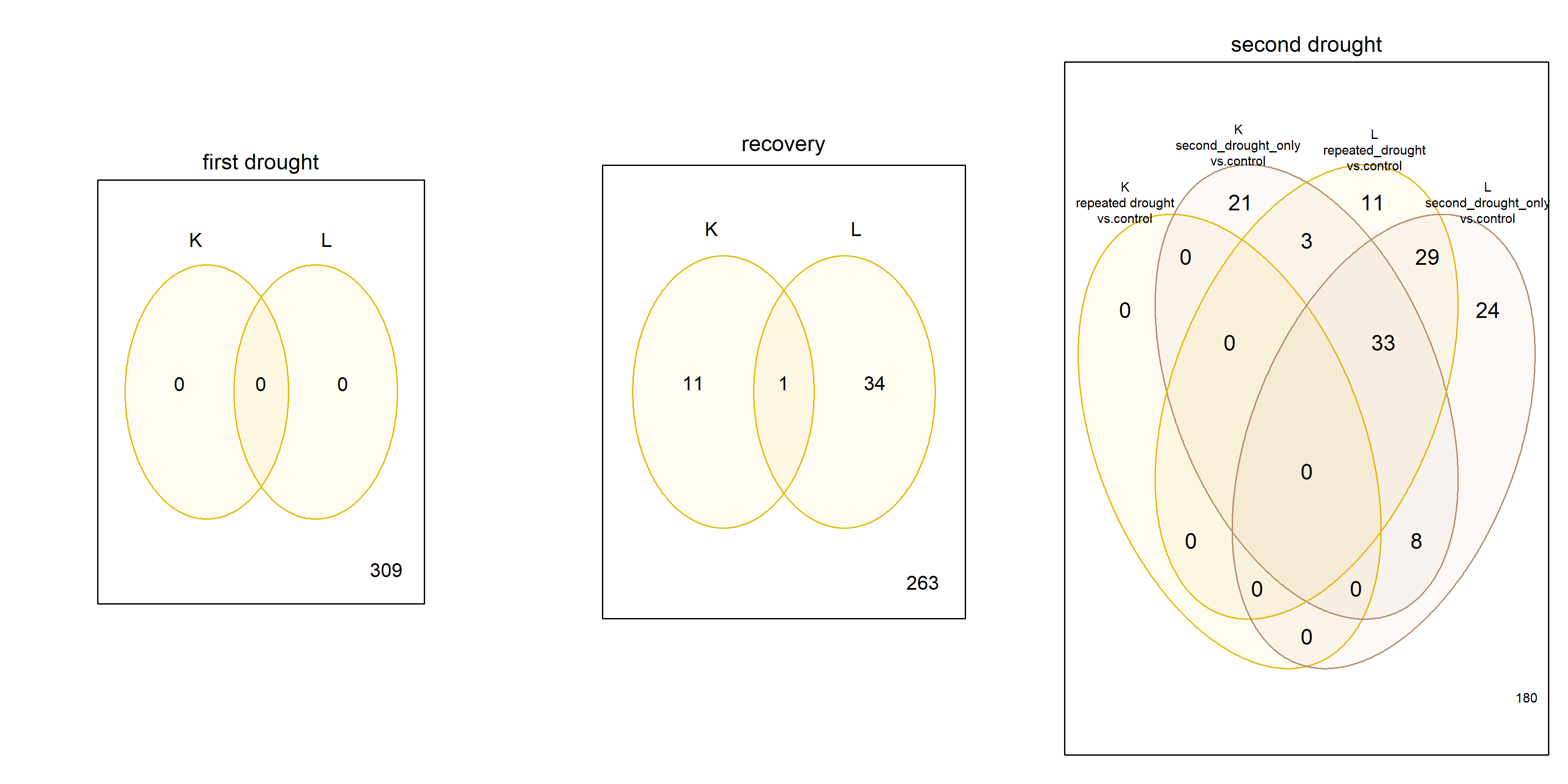

Supplement: Supplementary Figure 6 — Venn diagrams for lipid profile statistics at p.adj < 0.05. [file Image_6.tiff]

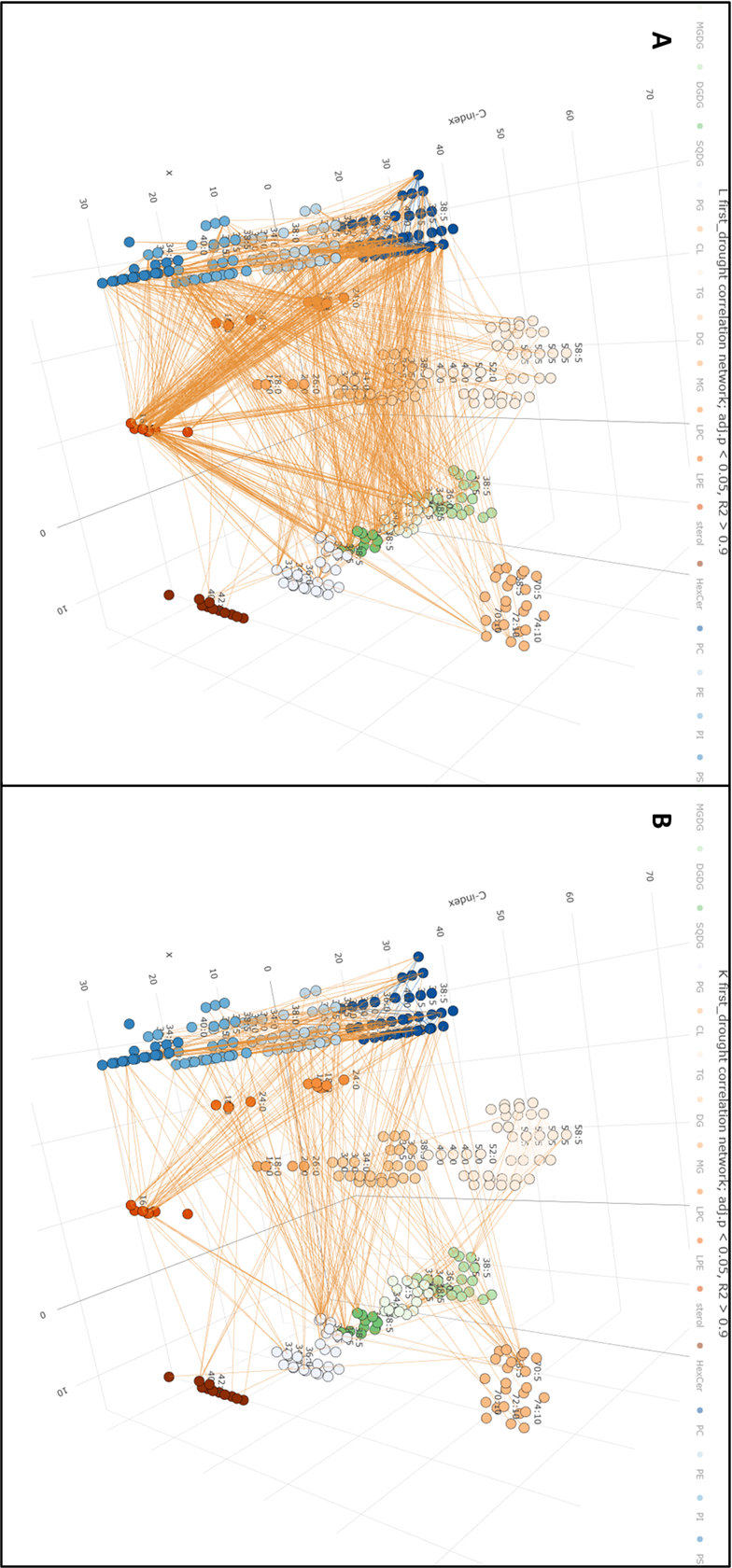

Supplement: Supplementary Figure 7 — Correlation network of lipids in hybrid LK (A) and hybrid KL (B) after the first drought event. The orange edges represent correlations (p.adj < 0.05, R² > 0.9) between different lipid classes. Correlations between lipids of the same class share the same edge and node colors. C-indices and double bonds are annotated for 0, 5 and 10 double bonds. [file Image_7.tif]

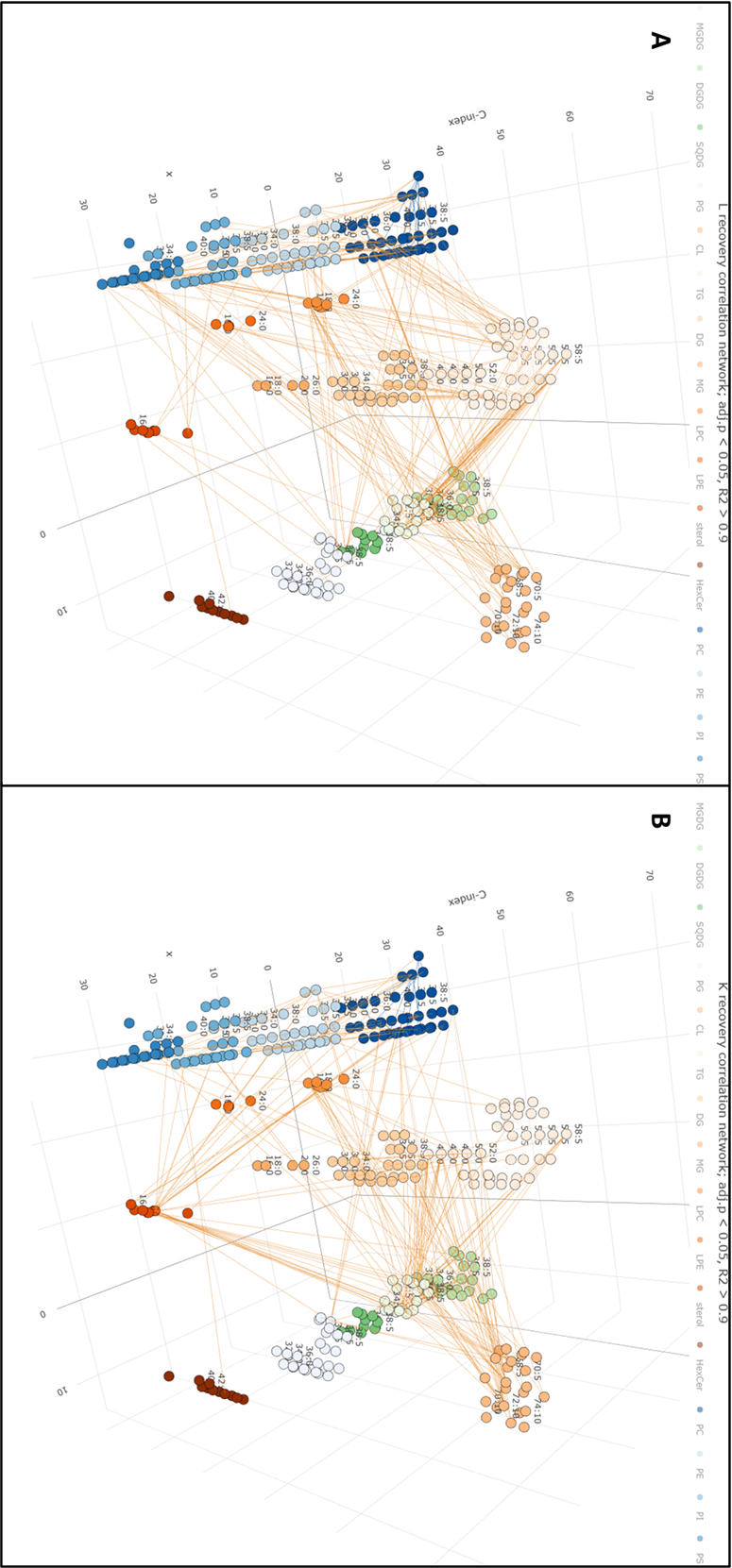

Supplement: Supplementary Figure 8 — Correlation network of lipids in hybrid LK (A) and hybrid KL (B) after recovery. The orange edges represent correlations (p.adj < 0.05, R² > 0.9) between different lipid classes. Correlations between lipids of the same class share the same edge and node colors. C-indices and double bonds are annotated for 0, 5 and 10 double bonds. [file Image_8.tif]

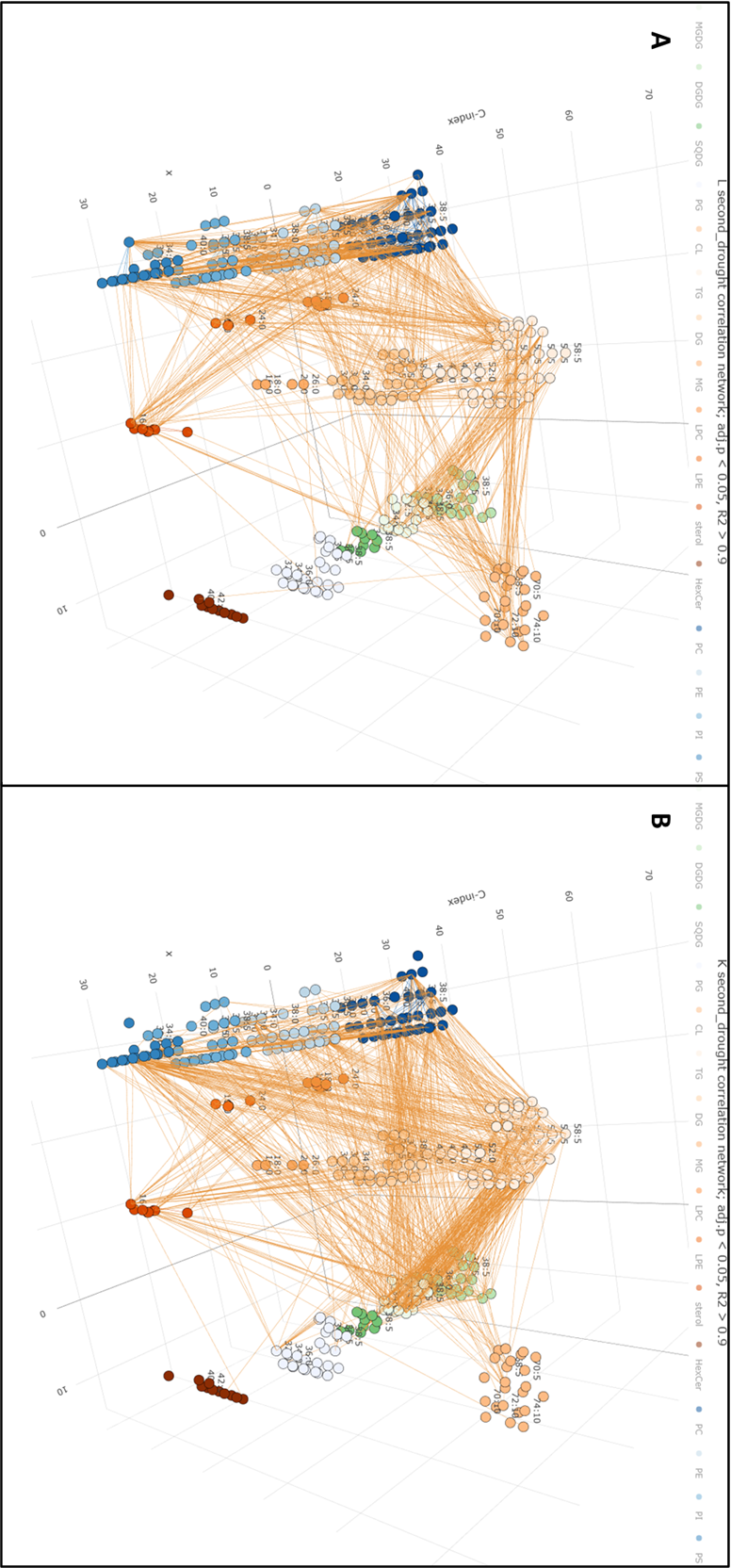

Supplement: Supplementary Figure 9 — Correlation network of lipids in hybrid LK (A) and hybrid KL (B) after second drought in the repeated drought treatments. The orange edges represent correlations (p.adj < 0.05, R² > 0.9) between different lipid classes. Correlations between lipids of the same class share the same edge and node colors. C-indices and double bonds are annotated for 0, 5 and 10 double bonds. [file Image_9.tif]

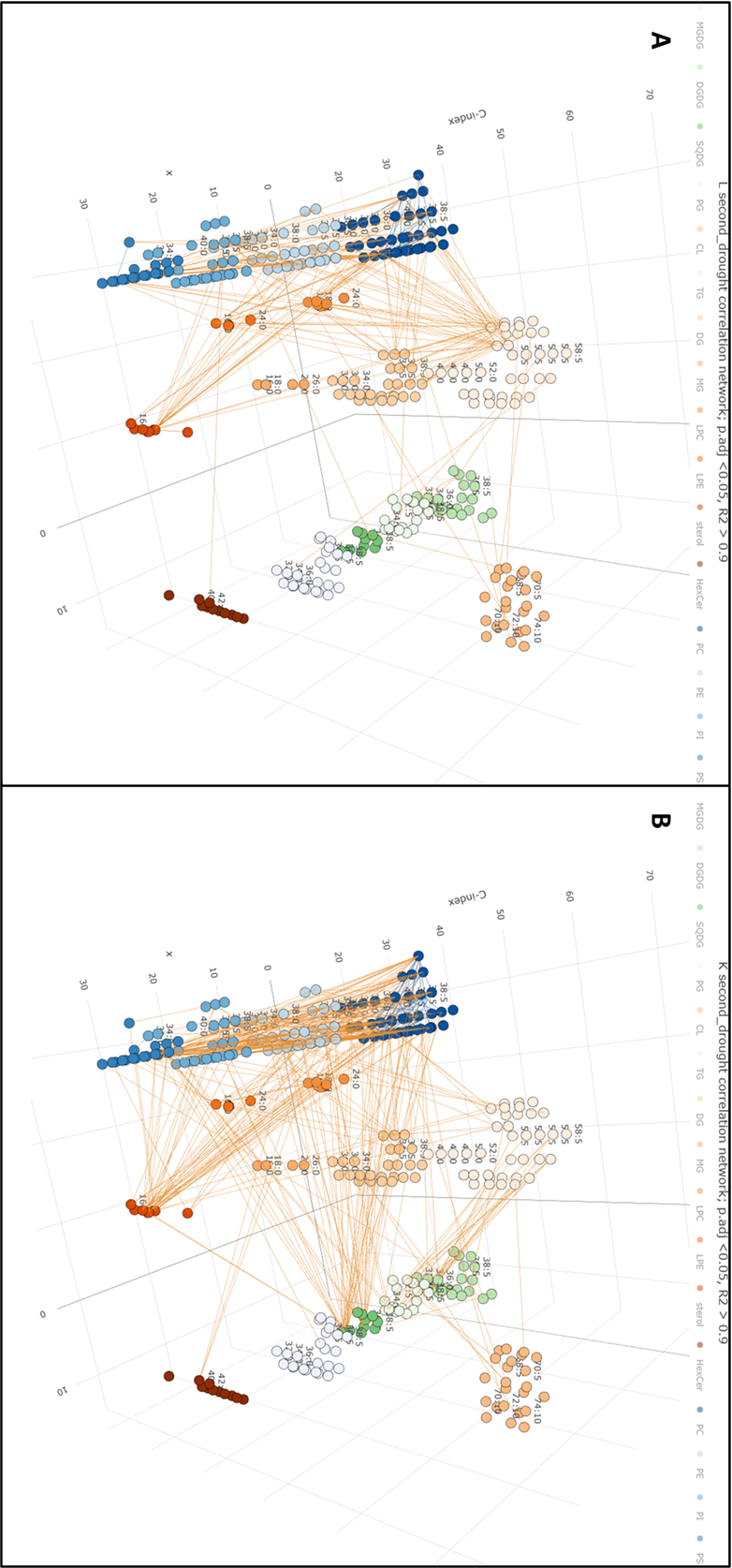

Supplement: Supplementary Figure 10 — Correlation network of lipids in hybrid K L (A) and hybrid L K (B) after second drought in the second drought only treatments. The orange edges represent correlations (p.adj < 0.05, R² > 0.9) between different lipid classes. Correlations between lipids of the same class share the same edge and node colors. C-indices and double bonds are annotated for 0, 5 and 10 double bonds. [file Image_10.tif]
